# A Highly Active Porous Mo_2_C-Mo_2_N Heterostructure on Carbon Nanowalls/Diamond for a High-Current Hydrogen Evolution Reaction

**DOI:** 10.3390/nano14030243

**Published:** 2024-01-23

**Authors:** Zhaofeng Zhai, Chuyan Zhang, Bin Chen, Lusheng Liu, Haozhe Song, Bing Yang, Ziwen Zheng, Junyao Li, Xin Jiang, Nan Huang

**Affiliations:** 1 Shenyang National Laboratory for Materials Science (SYNL), Institute of Metal Research (IMR), Chinese Academy of Sciences (CAS), No. 72 Wenhua Road, Shenyang 110016, China; zfzhai@imr.ac.cn (Z.Z.); zhangcy@imr.ac.cn (C.Z.); bchen18s@imr.ac.cn (B.C.); lsliu@imr.ac.cn (L.L.); hzsong@imr.ac.cn (H.S.); byang@imr.ac.cn (B.Y.); zwzheng21s@imr.ac.cn (Z.Z.); ljy2464011899@163.com (J.L.); 2 School of Materials Science and Engineering, University of Science and Technology of China, No. 72 Wenhua Road, Shenyang 110016, China; 3 Institute of Materials Engineering, University of Siegen, No. 9-11 Paul-Bonatz-Str., 57076 Siegen, Germany

**Keywords:** hydrogen evolution reaction, high current density, carbon nanowalls, diamond, Mo_2_C, Mo_2_N, heterostructure

## Abstract

Developing non-precious metal-based electrocatalysts operating in high-current densities is highly demanded for the industry-level electrochemical hydrogen evolution reaction (HER). Here, we report the facile preparation of binder-free Mo_2_C-Mo_2_N heterostructures on carbon nanowalls/diamond (CNWs/D) via ultrasonic soaking followed by an annealing treatment. The experimental investigations and density functional theory calculations reveal the downshift of the d-band center caused by the heterojunction between Mo_2_C/Mo_2_N triggering highly active interfacial sites with a nearly zero ∆*G*_H*_ value. Furthermore, the 3D-networked CNWs/D, as the current collector, features high electrical conductivity and large surface area, greatly boosting the electron transfer rate of HER occurring on the interfacial sites of Mo_2_C-Mo_2_N. Consequently, the self-supporting Mo_2_C-Mo_2_N@CNWs/D exhibits significantly low overpotentials of 137.8 and 194.4 mV at high current densities of 500 and 1000 mA/cm^2^, respectively, in an alkaline solution, which far surpass the benchmark Pt/C (228.5 and 359.3 mV) and are superior to most transition-metal-based materials. This work presents a cost-effective and high-efficiency non-precious metal-based electrocatalyst candidate for the electrochemical hydrogen production industry.

## 1. Introduction

Hydrogen energy, characterized by cleanliness and high energy density, is emerging as a sustainable alternative to fossil fuels [1,2]. The electrochemical hydrogen evolution reaction (HER) holds significant promise for large-area green hydrogen production, offering a solution to the high carbon emissions associated with the traditional steam reforming technique, due to the advantages of utilizing an exhaustless and cost-effective water resource, zero carbon emission, and ensuring high purity (>95%) [3,4,5]. Achieving this industrial goal requires highly active and inexpensive HER electrocatalysts capable of operating at high current density (≥500 mA/cm^2^) [1,6,7]. While Pt-based materials are regarded as excellent HER electrocatalysts, they suffer from the penalty of scarcity and a high cost [8]. Therefore, it is highly required to develop efficient non-precious metal alternatives for high-current HER applications.

As non-precious-metal electrocatalysts, Mo_2_C-based nanostructures have provoked significant attention from researchers due to their earth abundance, low cost, analogous d-orbital electronic structure to the Pt, and good resistance to dissolution [9,10,11]. However, the high d-band center position of the Mo site in the Mo_2_C induces a strong hydrogen-binding that is detrimental to the desorption process and consequently resulting in an unsatisfactory HER activity [12,13]. For instance, Chi et al. demonstrated that a large overpotential of 189 mV is required for the Mo_2_C nanospheres to reach a current density of 10 mA/cm^2^ in 1 M KOH [14]. To overcome the sluggish reaction kinetics, heteroatom doping with either nonmetals, such as N [15] and P [16], or metals, such as Ni [17] and Co [18], into the Mo_2_C as well as the design of heterostructures [19,20] have been employed to create a modulation of the electronic structure and boost the intrinsic HER activity. In this context, a Mo_2_C-Mo_2_N heterostructure was prepared by calcining Mo-containing polyoxometalates and graphene oxide in a NH_3_ atmosphere and exhibited an optimized overpotential of 154 mV in 1 M KOH [21]. Recently, a lower overpotential of 88.1 mV at 10 mA/cm^2^ was reported based on a ZIF-67-derived Mo_2_C-Mo_2_N catalyst [22]. It is verified that the construction of a heterostructure is a viable and efficient way to improve HER performance. Nevertheless, most studies focus on the HER characteristics at low current density, e.g., 10 and 100 mA/cm^2^, which falls short of addressing the practical application at high current operation [23,24]. In addition, in the case of above nanoparticle-type electrocatalysts, binders were needed to immobilize the particles onto a current collector, which possesses, however, several drawbacks, such as restricting exposure of the active sites, impeding mass transport, and obstructing electron transfer that reduce the overall HER performance [25,26].

In this study, we prepared binder-free porous Mo_2_C-Mo_2_N heterostructures on the carbon-nanowalls/diamond-coated carbon cloth (Mo_2_C-Mo_2_N@CNWs/D) via facile soaking in an ammonium molybdate solution followed by annealing with melamine. In this self-supporting electrocatalyst, the carbon nanowalls/diamond (CNWs/D), with a high electrical conductivity, open accessible surface, and large specific area, serves as a template for depositing Mo_2_C-Mo_2_N, efficiently enlarging the electrically active surface area (ECSA), as well as a current collector, greatly facilitating the electron transfer during HER. An electrochemically active Mo_2_C-Mo_2_N heterostructure is finely tuned in phase constituents to attain optimum HER characteristics. Density functional theory (DFT) calculations further reveal that the atoms located at the Mo_2_C-Mo_2_N interface possess greatly enhanced intrinsic activity. Derived from the excellent current collector and the boosted intrinsic activity, consequently, much lower overpotentials at both low (10 mA/cm^2^) and high current densities (500 and 1000 mA/cm^2^) are achieved on Mo_2_C-Mo_2_N@CNWs/D in 1 M KOH. This work provides a facile methodology for the construction of a non-precious electrocatalyst and promising applications in electrochemical high-current hydrogen production.

## 2. Materials and Methods

### 2.1. Material Preparation

CNW_S_/D film was prepared on carbon cloth (CC) using microwave plasma chemical vapor deposition (MPCVD) with a 915 MHz reactor (Cyrannus, Iplas Innovative Plasma Systems GmbH, Troisdorf, Germany). The CC was ultrasonically cleaned in acetone, ethanol, water, and nitric acid, followed by being ultrasonically seeded in the diamond suspension for 30 min. It was verified that the diamond seed enhanced the growth rate and robustness of carbon nanowalls (CNWs). The seeded CC was then placed onto the Al_2_O_3_ holder in the MPCVD chamber. During the deposition, a microwave power of 6 kW, a H_2_ flow rate of 200 sccm, a CH_4_ flow rate of 14 sccm, and a chamber pressure of ~30 mbar were employed. The substrate temperature was estimated to be ~1065 °C, monitored using an infrared pyrometer. After 60 min of growth, the plasma was shut down and the pristine CNWs/D film was obtained on the CC.

The preparation of Mo_2_C-Mo_2_N@CNW_S_/D involved soaking CNW_S_/D in a Mo-containing solution and thermal treatment with melamine. Before the soaking, the pristine CNW_S_/D film surface was modified to become O-terminated through UV irradiation in the air, in order to impart a hydrophilic property in an aqueous solution. After soaking for 30 min in an ultrasonic bath made up of 31.39 wt% (NH_4_)_6_Mo_7_O_24_ (Sinopharm Chemical Reagent Co., Ltd., AR, Shanghai, China), the obtained Mo precursor@CNW_S_/D was annealed with 0.2 of melamine (Aladdin, 99%) positioned upstream at a heat rate of 2 °C/min in an Ar/H_2_ atmosphere. According to the annealing temperature (including 500, 650, 700, 750, 800, and 850 °C), the obtained electrocatalysts are designated as Mo_2_C-Mo_2_N@CNW_S_/D-500, -650, -700, -750, -800, and -850, respectively. For comparison, Mo_2_C@CNW_S_/D-650 was prepared using the same routine at 650 °C, albeit without the introduction of melamine. Mo_2_C-Mo_2_N@CC-650 was prepared on CC using a similar routine at 650 °C.

### 2.2. Material Characterization

The morphology and elemental composition were investigated by using a field-emission scanning electron microscope (SEM, Hitachi, SU 70, Tokyo, Japan). The micro-XRD (Bruker, D8 Discover, Billerica, MA, USA) using a Co Kα_1_ radiation source (λ = 1.78897 Å) was employed to obtain the phase information. In addition, the carbon phase of CNW_S_/D was studied using a Raman spectroscope (Horiba, Labram HR Evolution instrument, Kyoto, Japan) based on a 532 nm laser. The microstructure and elemental mapping distribution were characterized by using a transmission electron microscope (TEM, ThermoFisher, Tablos F200X, Waltham, MA, USA) equipped with an energy dispersive X-ray spectrometer (EDS, ThermoFisher, Super X G2, Waltham, MA, USA).

To access the surficial chemical state, X-ray photoelectron spectra (XPS) were recorded by using a Thermo ESCALAB Xi^+^ instrument (Waltham, MA, USA) with an Al Kα source (*hυ* = 1486.6 eV). The bare surface of Mo_2_C-Mo_2_N@CNWs/D was measured without sputtering. In addition, during the XPS test, the power was 150 W, and the electron emission angle was 55°. The analyzed area had a diameter of 500 μm. The base pressure was 2.77 × 10^−7^ mbar. The charge neutralizer was used to reduce the possibility that the sample charges up. Furthermore, all the acquired XPS data were corrected based on the highest peak located at 284.6, assigned to the adventitious carbon contamination [27]. To reveal the chemical state of Mo, C, and N species of Mo_2_C-Mo_2_N@CNWs/D, peak fitting was conducted further using XPSPEAK software (Version 4.1) based on the acquired XPS spectra. All spectra were fitted with Shirley backgrounds and the Voigt function (80% Gaussian and 20% Lerentzian). For the Mo 3d fitting, the 3d_3/2_/3d_5/2_ area ratio was constrained at around 0.66. In addition, the XPS fitting conformed to the criterion of qualitative self-consistency [27].

### 2.3. Electrochemical Measurements

Electrocatalytic HER measurement of all samples was carried out on an electrochemical workstation (Metrohm, PGSTAT302N, Herisau, Switzerland) in a three-electrode cell at room temperature. The three-electrode system was composed of a self-supporting working electrode, a graphite rod counter electrode, and a Hg/HgO (1 M KOH) reference electrode. The measured potential was converted to a reversible hydrogen electrode (RHE) using the following equation:

*E*_RHE_ = *E*_Hg/HgO_ + 0.098 + 0.0591 × pH (1)

Linear sweep voltammetry (LSV) was carried out at the scan rate of 0.002 V/s to evaluate the overpotential and Tafel slope during the HER process. Cyclic voltammetry (CV) was performed at various scan rates ranging from 0.04 to 0.12 V/s to estimate the ECSA of the electrocatalyst. In addition, electrochemical impedance spectroscopy (EIS) was recorded at an overpotential of 74.6 mV with a 10 mV amplitude in the frequency range from 100 kHz to 0.1 Hz. Prior to the electrochemical test, Ar gas (≥99.999%) was bubbled into the electrolyte for 20 min to preclude the inference of the oxygen reduction reaction. All LSV potentials were demonstrated through a 95% *iR*-compensation based on the EIS results. All current densities were demonstrated after normalization using the geometric area of the electrocatalyst.

### 2.4. Calculation Method

DFT calculation was conducted using the plane-wave code implemented in the Vienna Ab Initio Simulation Package (VASP). The electron exchange and correlation are described with the generalized gradient approximation as parameterized by Perdew, Burke, and Ernzerhof (GGA-PBE), and the interaction between ions and electrons is described using the projector augmented wave method (PAW). The effective valence used here of each atom is 4.000 for the C atom, 5.000 for the N atom, and 6.000 for the Mo atom, respectively. For all calculations, the kinetic cut-off energy of 400 eV with the self-consistent field (SCF) tolerance of 1 × 10^−6^ eV and 0.02 eV Å^−1^ was adopted. Figure S1 displays the side-view and top-view of the constructed model structure. Typically, the (111) crystal surface of Mo_2_N and the (101) crystal surface of Mo_2_C were chosen, consistent with the TEM and XRD results. To model the Mo_2_C-Mo_2_N interface structure, we constructed firstly a supercell consisting of 2 × 3 Mo_2_C (101) unit cells with 2 × 2 Mo_2_N (111) unit cells in the vertical direction with a lattice mismatch of 0.3%. Then, the supercell was rotated 90° to expose the interface sites for subsequent adsorption energy calculations. A vacuum region of 20 Å was set along the z direction to avoid the interaction between slabs. For the balance of the calculation accuracy and time cost, the k-point mesh was employed as 3 × 3 × 1 k-points for the structural relaxation and 9 × 9 × 1 for the energy band.

After the structural optimization, the adsorption free energy differences of H^*^ (∆*G*_H*_) on different surface sites were determined as

∆*G*_H*_ = ∆*E*_H*_ + ∆*ZPE* − T∆*S* (2)

where ∆*E*_H*_ is the energy change during the H adsorption/desorption process. ∆*ZPE* and ∆*S* are the difference in zero-point energy and entropy, respectively. T is the system temperature (298.15 K, in our calculation). For H* on different surfaces sites, all 3N degrees of freedom are treated as vibrational motions while neglecting the contributions from the material surfaces. The Δ*G*_H*_ was calculated using *Vaspkit* software (Version 1.4.1) from Δ*E*_H*_, temperature, pressure, and calculated vibrational energy [28].

## 3. Results and Discussion

### 3.1. Microstructure Characterizations

**Figure 1:**
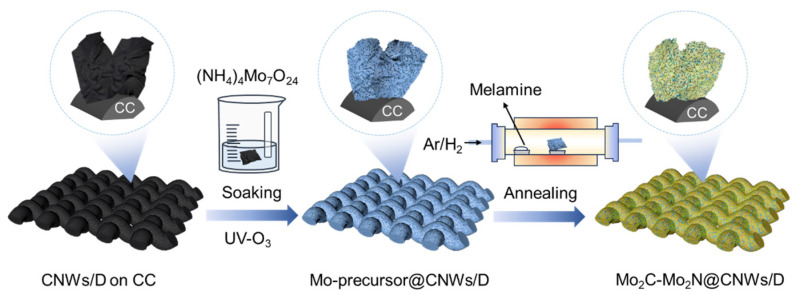
Schemic illustration for the preparation of Mo_2_C-Mo_2_N@CNWs/D.

Figure 1 schematically shows the synthesis route of the Mo_2_C-Mo_2_N heterostructure on the CNW/D-coated CC. Typically, the CNW_S_/D was initially deposited on the CC using the MPCVD system, followed by ultrasonic soaking in a (NH_4_)_2_MoO_4_ aqueous solution and then drying through centrifugation. Afterwards, the obtained Mo precursor@CNW_S_/D was immediately calcined with melamine in the Ar/H_2_ atmosphere. Finally, the Mo_2_C-Mo_2_N@CNW_S_/D was prepared. It is noteworthy that the proportion of Mo_2_C to Mo_2_N could be manipulated by varying the calcination temperature.

**Figure 2:**
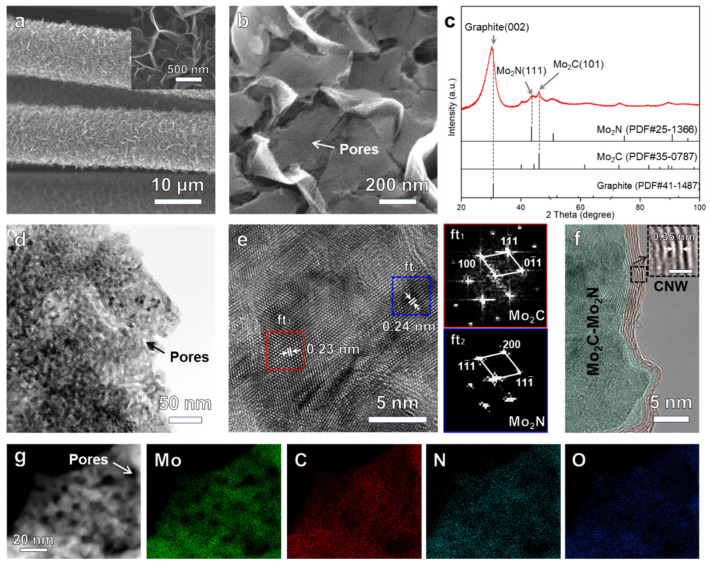
Microstructure characterizations of Mo_2_C-Mo_2_N@CNWs/D. (**a**) SEM image of the pristine CNW/D-coated CC. (**b**) SEM image and (**c**) XRD pattern of the Mo_2_C-Mo_2_N@CNWs/D. (**d**) Low-magnification and (**e**,**f**) high-resolution TEM images of Mo_2_C-Mo_2_N@CNWs/D. (**g**) HAADF TEM image and corresponding EDS elemental mapping images for Mo, C, N, and O of the Mo_2_C-Mo_2_N@CNWs/D.

Figure 2a demonstrates the morphology of the CNW/D-coated CC. Compared to the smooth surface of bare CC (see Figure S2), the CNW/D-coated CC is completed surrounded by a furry 3D-networked nanowall after MPCVD. The magnified inset shows the nanowall almost vertically aligned on the CC. In the Raman spectrum (Figure S3), well-separated peaks of the D band positioning at ~1344 cm^−1^, the G band at ~1566 cm^−1^, the D’ band at ~1615 cm^−1^, as well as the outstanding 2D band at ~2700 cm^−1^ are observed, and the *I*_D_/*I*_G_ ratio is estimated to be 0.34. The *I*_D_/*I*_G_ value is lower than the reported carbon nanofiber and highly branched graphene nanosheets [29,30], suggesting low-disordered CNWs were prepared on the CC substrate [31]. Figure 2b–g show the microstructure and constituent of the Mo_2_C-Mo_2_N@CNW_S_/D after calcination. Notably, porous nanostructures completely cover the CNW_S_/D (see Figure 2b). The CNWs/D, with an open accessible surface and large specific area, works as a great nano-template for the conformal growth of Mo_2_C-Mo_2_N. XRD measurement was used to determine the phases. As illustrated in Figure 2c, besides the predominated graphite peak, one can see two major characteristic peaks at 44° and 46°, matching well with the (111) plane of Mo_2_N (PDF#25-1366) and (101) of Mo_2_C (PDF#35-0787), respectively. This distinctly verifies that the Mo_2_C-Mo_2_N composite was rationally synthesized. In order to access deep insight into the microstructure, TEM characterizations were carried out on the Mo_2_C-Mo_2_N@CNWs/D. Figure 2d illustrates that Mo_2_C-Mo_2_N has a porous structure. The pore size was estimated to be less than 10 nm, which can be clearly observed in the high-angle annular dark field (HAADF) TEM image in Figure S4. Figure 2e shows the high-resolution TEM image of Mo_2_C-Mo_2_N. As exhibited by the blue rectangle, the distances between adjacent lattice planes are measured to be 0.24 nm, consistent with the theoretical d-spacing of the {111} plane of Mo_2_N. The fast-Fourier transformation pattern in the bottom-right panel also matches well with the Mo_2_N diffracting spots taken along the [011] zone axis. Such results suggest that the Mo_2_N (PDF#25-1366) phase constitutes the region ft_2_. In addition, the measured interlayer spacing of 0.23 nm corresponding to the {101} plane of Mo_2_C and the ft_1_ pattern related to the theoretical Mo_2_C spots along the [0$\overset{-}{1}\overset{-}{1}$] zone indicate that the Mo_2_C (PDF#35-0787) phase is obtained in the region ft_2_. Importantly, an intimate interface is observed at the transition zone, hinting that a heterointerface is created between Mo_2_N and Mo_2_C. Due to the crystal lattice misfit, lattice distortion occurs at the heterointerface. It is believed that such distortion should induce an alteration in the local electronic structure, which accordingly manages the activity toward HER [32]. In addition, the CNWs, also designated as multilayered graphene with a lattice spacing of 0.35 nm, are obviously distinguished in the high-resolution TEM image taken near the edges (see Figure 2f). As an excellent current collector, CNWs support the Mo_2_C-Mo_2_N heterostructure, endowing high electrical conductivity to facilitate the transport of electrons generated during the HER into the electrochemical loop. Moreover, as shown in Figure 2g and Figure S5, the HAADF image and corresponding EDS mapping images display a nearly even distribution of Mo, C, and N elements within the porous structure, suggesting the formation of abundant heterostructures in the catalyst.

All of the above results reveal that the porous Mo_2_C-Mo_2_N heterostructure was facilely fabricated on the CNW_S_/D. Accounting for abundant active sites from porous Mo_2_C-Mo_2_N heterostructures and an excellent current collector from CNWs, an outstanding high-current HER performance is foreseen.

### 3.2. Performance of Hydrogen Evolution Reaction

**Figure 3:**
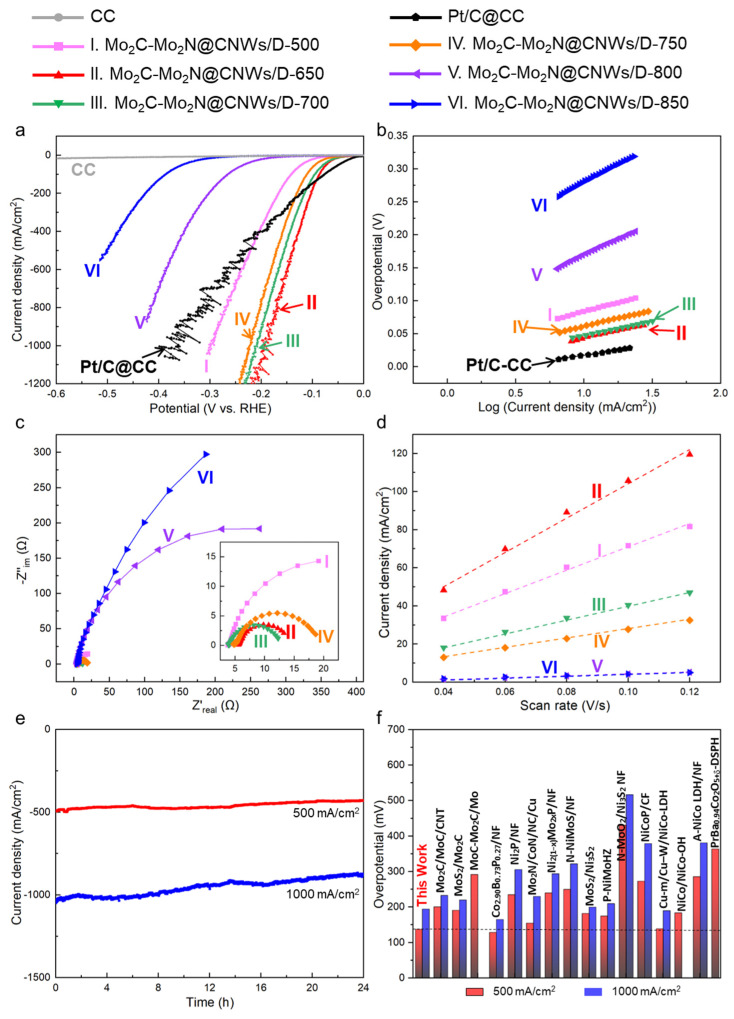
Electrocatalytic HER performance of Mo_2_C-Mo_2_N@CNWs/D in 1 M KOH. (**a**) Polarization curves and corresponding (**b**) Tafel plots. (**c**) Nyquist plots, and the inset showcases the Nyquist plots at high frequency. (**d**) Capacitive current variation as a function of scan rate from 0.04 to 0.12 V/s. (**e**) Long-term HER durability test of Mo_2_C-Mo_2_N@CNWs/D-650 at high current densities. (**f**) Statistics histogram for the overpotential of our prepared Mo_2_C-Mo_2_N@CNWs/D and previously reported transition-metal-based electrocatalysts at 500 and 1000 mA/cm^2^. The curves of CC and Pt/C@CC catalyst are demonstrated in (**a**,**b**) as control.

The HER performance of the obtained Mo_2_C-Mo_2_N@CNW_S_/D catalyst was investigated in 1 M KOH based on a typical three-electrode system. Figure S6 shows that the Mo_2_C-Mo_2_N@CNWs/D prepared with the annealing time of 180 min demonstrates a better HER performance at high current densities than those with annealing times of 60 and 180 min. In the following, the effect of annealing temperature on the HER performance is investigated further. For comparison, the performance of the Pt/C@CC catalyst (2.0 mg/cm^2^) prepared using the drop-casting method and CC was also studied. As depicted in Figure 3a, the electrocatalytic performance of CC is very poor. Pt/C exhibits a high HER activity with an overpotential (*η*_10_) of 16.8 mV at a low current density of 10 mA/cm^2^. Comparatively, the *η*_10_ are measured to be 83.7, 42.8, 47.9, 62.6, 169.3, and 281.7 mV for the Mo_2_C-Mo_2_N@CNW_S_/D-500, -650, -700, -750, -800, and -850. Table 1 lists the HER characteristics of these catalysts. Note that the overpotential gradually declines as the annealed temperature increases, reaches the minimum point of 42.8 mV for Mo_2_C-Mo_2_N@CNW_S_/D-650, and then goes up with elevating the temperature further.

To unveil the rate-determining step of the HER process, Figure 3b shows the Tafel plots derived from the LSV data. Pt/C@CC possesses the smallest Tafel slope estimated to be 32.5 mV/dec, consistent with the previously reported value of 31.0 mV/dec [33]. As the temperature increases, electrocatalysts demonstrate a similar variation trend in the Tafel slope as observed in the overpotential. As listed in Table 1, Mo_2_C-Mo_2_N@CNW_S_/D-650 has the smallest Tafel slope estimated to be 45.6 mV/dec. Generally, the HER occurring on the electrocatalyst involves the electrochemical adsorption of hydrogen (Volmer reaction), followed by either the electrochemical desorption (Heyrovsky reaction) or the chemical desorption (Tafel reaction) [34]. Given the Tafel slope of 120 mV/dec for the Volmer reaction-determining step, 30 mV/dec for the Tafel reaction, and 40 mV/dec for the Heyrovsky reaction, the HER mechanism closely adheres to the Heyrovsky mechanism on the Mo_2_C-Mo_2_N@CNW_S_/D-650. In contrast, other obtained electrocatalysts exhibit a mixed Volmer–Heyrovsky mechanism.

To gain a comprehensive understanding of the charge transfer kinetics during the HER, EIS tests were carried out, and the Nyquist plots were recorded in Figure 3c. The curves are composed of a semicircle related to the electron transfer behavior on the active sites of the electrode [35]. The Mo_2_C-Mo_2_N@CNW_S_/D-650 demonstrates the smallest semicircle, and the charge transfer resistance (*R*_ct_) is calculated to be 8.59 Ω, derived from the fitting data using the Randles equivalent circuit (see Table 1). In addition, the ECSA of Mo_2_C-Mo_2_N@CNW_S_/D was estimated through the electrochemical double-layer capacitance tests at different scan rates. As shown in Figure S7, no faradaic features are observed in these CV curves. Correspondingly, the capacitive current densities at ~0.23 V were plotted as a function of the scan rate in Figure 3d. The electrochemical double-layer capacitance of Mo_2_C-Mo_2_N@CNW_S_/D-650 is the highest at 891 mF/cm^2^, which then quickly decreases with the increase in the temperature.

Furthermore, the stability of Mo_2_C-Mo_2_N@CNWs/D-650 was determined through 24 h HER operation at high current densities. Figure 3e shows that the current density could retain 86.7% and 83.2% of the initial values at 500 and 1000 mA/cm^2^, respectively, suggesting the good stability of the Mo_2_C-Mo_2_N@CNWs/D electrocatalyst. In addition, Mo_2_C-Mo_2_N@CNWs/D-650 exhibits minor structural variations after 24 h HER operation at 500 mA/cm^2^, which implies the good structural stability (see Figure S8).

The above results demonstrate that the Mo_2_C-Mo_2_N@CNW_S_/D-650 exhibits the best performance, that is, the lowest overpotential (*η*_10_ = 42.8 mV), the lowest Tafel slope (45.6 mV/dec), the lowest *R*_ct_ (8.59 Ω), and the largest capacitance (891 mF/cm^2^), among our prepared electrocatalysts. Furthermore, HER performances of the Mo_2_C-Mo_2_N@CNW_S_/D-650 and other reported transition-metal-based electrocatalysts are compared in Table 2. The *η*_10_ value is smaller than that of the reported molybdenum-based materials, such as Mo_2_C-Mo_2_N/HGr [21], Mo*_x_*C [13], P-MoP/Mo_2_N [36], Mo_2_C/MoC/CNT [20], and MoC-Mo_2_C/Mo [37]. More impressively, the Mo_2_C-Mo_2_N@CNW_S_/D-650 desires overpotentials of only 137.8 and 194.4 mV to achieve high current densities of 500 and 1000 mA/cm^2^, respectively. These values are much lower than those of benchmark Pt/C@CC (*η*_500_ = 228.5 mV, *η*_1000_ = 359.3 mV). Actually, Figure 3f and Table 2 verify that the Mo_2_C-Mo_2_N@CNW_S_/D-650 stands out as one of the most highly active electrocatalysts at high current density (≥500 mA/cm^2^) in alkaline HER, compared with previously reported transition-metal-based materials [19,20,26,33,37,38,39,40,41,42,43,44,45,46,47,48].

### 3.3. Dependance of Hydrogen Evolution Reaction Performance on the Surface Chemical State

It is noteworthy that the electrocatalytic properties could be facilely modulated by tuning the calcination temperature. As the temperature increases from 500 to 650 °C, the overpotential, Tafel slope, and *R*_ct_ of the catalysts demonstrate a decreasing trend. With elevating the temperature further until 850 °C, these HER characteristics all quickly increase.

**Figure 4:**
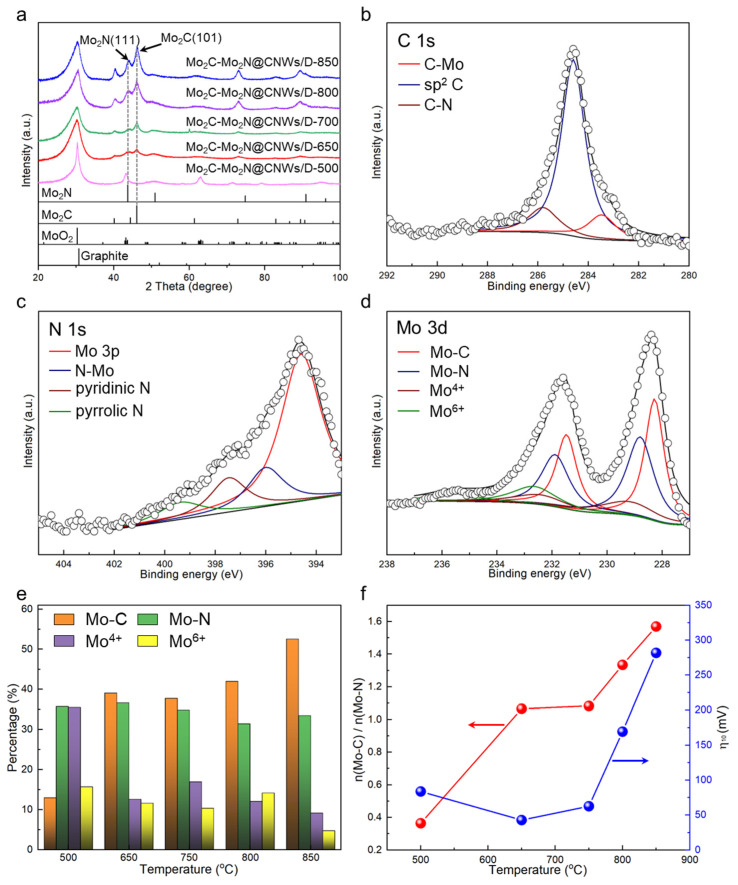
Constituent characterizations of Mo_2_C-Mo_2_N@CNWs/D. (**a**) XRD patterns of Mo_2_C-Mo_2_N@CNWs/D-500, -650, -700, -800, and -850. High-resolution XPS spectra and deconvoluted plots of (**b**) C 1s, (**c**) N 1s, and (**d**) Mo 3d in Mo_2_C-Mo_2_N@CNWs/D-650. (**e**) Histogram illustrating the statistical distribution of Mo species percentages and (**f**) the ratio of *n*(Mo-C)/*n*(Mo-N) derived from XPS deconvolution as a function of the temperature. The *η*_10_ variation with the temperature is also depicted in panel (**f**).

To deeply understand such variation, the crystal phase and surface chemical state of Mo_2_C-Mo_2_N@CNW_S_/D are thoroughly studied. Figure S9 shows that the *I*_D_/*I*_G_ of Mo_2_C-Mo_2_N@CNWs/D is estimated to be 0.53, slightly larger than that of bare CNWs/D. This indicates that defects are created in the CNWs through annealing treatment. The *I*_D_/*I*_G_ value does not vary significantly as the annealing temperature increases. Figure 4a displays the XRD patterns of the catalysts prepared at different temperatures. The Mo_2_C-Mo_2_N@CNW_S_/D-500 sample shows a prominent peak positioned at ~43° and a feeble peak at 51°, corresponding to ($\overset{-}{2}$02)/(020) of MoO_2_ (PDF#73-1249) and (200) of Mo_2_N, respectively. Moving to 650^o^ or above, distinct peaks associated with the Mo_2_C (101) and Mo_2_N (111) planes are clearly seen. The intensity ratio of the Mo_2_C (101) peak to Mo_2_N (111) peak is further calculated, and the results are recorded in Figure S10. Mo_2_C-Mo_2_N@CNW_S_/D-650 shows a ratio of 1.15, which then gradually rises with the increase in annealing temperature. It is deduced that Mo_2_N is easily fabricated at a low temperature. When a higher temperature was employed, more Mo_2_C was obtained in the Mo_2_C-Mo_2_N@CNW_S_/D.

The surface chemical states could play a crucial role in the HER as the electrochemical reaction primarily occurs at the surface sites. Figure S11 shows the XPS survey spectrum of Mo_2_C-Mo_2_N@CNW_S_/D-650, and the signals from C, N, Mo, and O are clearly distinguished. In comparison to the XPS survey before the HER test, all elements remain at the surface of Mo_2_C-Mo_2_N@CNWs/D-650 after the HER test (see Figure S12). This indicates a good chemical stability of the prepared electrocatalyst. Moreover, the high-resolution XPS spectra of C 1s, N 1s, and Mo 3d are devaluated and scrutinized in Figure 4b–d. In the C 1s spectrum, three peaks located at a binding energy of 283.47, 284.60, and 285.80 eV are attributed to C-Mo bonds in Mo_2_C, sp^2^-carbon bonds, and C-N bonds, respectively (see Figure 4b) [34,49]. The C-Mo species constitute 12.1 at% of the C species on the surface. The N 1s spectrum was deconvoluted into three components: the peak at 396.00 eV assigned to the N-Mo bond, the peak at 397.47 eV to pyridinic N, and the peak at 399.39 eV to pyrrolic N (see Figure 4c) [50]. The peak positioning at 394.60 eV is derived from Mo 3P bonds [21,50]. The N-Mo species make up 41.5 at% of the surface N species. It is noteworthy that the C 1s and N 1s investigations indicate the coexistence of Mo-C and Mo-N bonds on the surface of the catalyst. This is further confirmed by the high-resolution Mo 3d spectrum shown in Figure 4d. The Mo 3d spectrum reveals four deconvoluted doublets: peaks at 228.30 and 231.48 eV corresponding to the Mo-C bonds, peaks at 228.80 and 231.89 eV to the Mo-N bonds, peaks at 229.20 and 232.45 eV to the Mo^4+^ species, and peaks at 232.65 and 235.56 eV to the Mo^6+^ species [37,50,51,52,53]. The Mo^4+^ and Mo^6+^ peaks are associated with MoO_2_ and MoO_3_, which stem from the unavoidable surface oxidation occurring when exposed to air [54]. In addition, the Mo 3d XPS spectra of Mo_2_C-Mo_2_N@CNW_S_/D-500, -750, -800, and -850 were recorded and deconvoluted using the similar doublets, as illustrated in Figure S13. Figure 4e presents a summary of the statistical percentage distribution of Mo species as the synthesis temperature varies. Mo_2_C-Mo_2_N@CNW_S_/D-500 possesses a higher percentage of Mo^4+^ species than others, aligning with the distinct MoO_2_ peak in the XRD result. Importantly, the percentage of the Mo-C bond generally increases from 13% to 53% with the temperature rising from 500 to 850 °C, while that of Mo-N bonds constantly remains around 33%. It is proposed that the high calcination temperature facilitates the transformation of the Mo-O (Mo^4+^ and Mo^6+^) bonds into Mo-C bonds. Keep in mind that the Mo-C and Mo-N bonds are regarded as the active sites for HER [55]. To reveal the relative variation of these bonds, the ratio changes in Mo-C content (*n*(Mo-C)) to Mo-N content (*n*(Mo-N)) were determined in Figure 4f. One can see that the ratio progressively increases as a function of temperature, well in line with the XRD investigations (see Figure 4a and Figure S10). Notably, it is the Mo_2_C-Mo_2_N@CNW_S_/D-650, with a ratio close to 1 between Mo-C and Mo-N bonds, that demonstrates the lowest HER overpotential (and best HER performance) among these catalysts.

**Figure 5:**
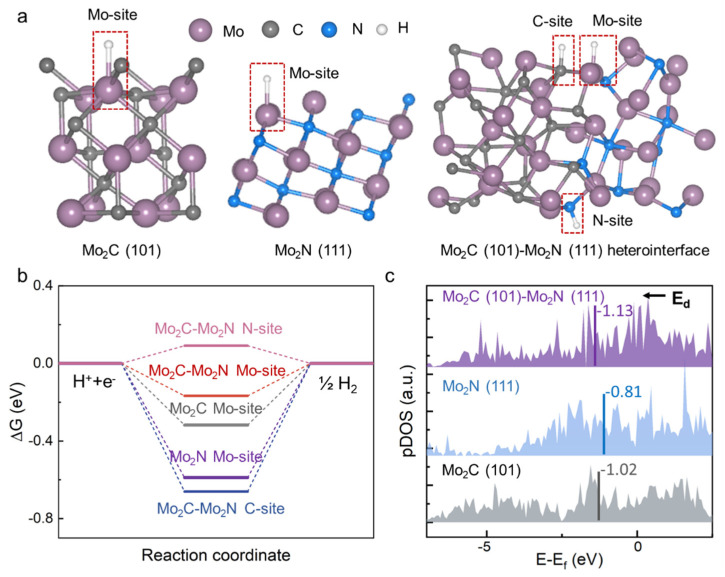
DFT calculations for HER energy variation and electronic states. (**a**) Optimized structural model with H^*^ adsorbable sites, (**b**) free energy diagram of H^*^ adsorption/desorption, and (**c**) partial density of states of Mo d orbitals for Mo_2_N (111) facet, Mo_2_C (101) facet, and Mo_2_C (101)-Mo_2_N (111) heterostructure. The Fermi level is referenced to 0 eV in panel (**c**).

To further explore the synergistic effects involved in the HER activity on the Mo_2_C-Mo_2_N heterostructure, DFT calculations were carried out. Figure 5a exhibits the optimized atomic model of the Mo_2_N(111), Mo_2_C (101), and Mo_2_N(111)/Mo_2_C(101) heterostructure consistently well with XRD and TEM results. The possible adsorption sites of H^*^ are also indicated in the model. Figure 5b displays the free energy diagram (∆*G*_H*_) of H^*^ adsorption/desorption on different electrocatalysts. ∆*G*_H*_ is supposed to be a significant indicator of the electrocatalyst activity. Regarding ideal ∆*G*_H*_, it is hoped that it is close to zero since either a negative ∆*G*_H*_, meaning the facile adsorption of H^*^ on the active site but challenging detachment, or a positive ∆*G*_H*_, indicating the arduous attachment of H on the active site but easy release, results in a large overpotential during the HER process [56]. As listed in Table S1, Mo_2_N and Mo_2_C have an exothermic ∆*G*_H*_, calculated to be −0.5889 and −0.3178 eV, respectively, implying an impediment to hydrogen release and hence a poor HER performance. For the Mo_2_C-Mo_2_N heterostructure, the ∆*G*_H*_ values for HER occurring on the interfacial N, Mo (bonded with N and C atoms), and C site are 0.0913, −0.1673, and −0.6604 eV, respectively. Therefore, interfacial N and Mo (bonded with N and C atoms) atoms are two major active centers for HER. More significantly, the |∆*G*_H*_| value of these centers is much closer to zero compared to those of Mo_2_N and Mo_2_C, which endows the desired balance between adsorption and desorption of H^*^, contributing to a high electrocatalytic activity of the Mo_2_C-Mo_2_N heterostructure. To support such theoretical results, Mo_2_C@CNW_S_/D-650 was prepared on the CC without the melamine introduction (see XRD pattern in Figure S14). Figure S15 and Table S2 confirm the beneficial effect derived from the Mo_2_C-Mo_2_N heterostructure on the HER, because the Mo_2_C-Mo_2_N@CNW_S_/D-650 possesses a much smaller overpotential (*η*_10_ = 42.8 mV) and lower Tafel slope (45.6 mV/dec) than the Mo_2_C@CNW_S_/D-650 (*η*_10_ = 107.9 mV, Tafel slope = 52.9 mV/dec).

To further explain the factors affecting the adsorption energy on the Mo_2_C-Mo_2_N heterostructure, we investigated the electronic states of Mo d orbitals and calculated the corresponding d-band center (Figure 5c). The heterostructure possesses a more negative d-band center (−1.13 eV) than that of Mo_2_N (−0.81 eV) and Mo_2_C (−1.02 eV), indicating that the surplus H^*^ binding capacity favorably weakens, which thus contributes to a decreased |∆*G*_H*_| value on the interfacial Mo site of Mo_2_C-Mo_2_N [57], as explained in Figure 5b. In addition, the local density of state of Mo_2_C-Mo_2_N is higher than that of Mo_2_N and Mo_2_C near the Fermi level. Such enhancement facilitates a rapid charge transfer rate at the electrolyte(H^+^)/electrocatalyst interface in the HER, consistently supported by the much lower *R*_ct_ on the Mo_2_C-Mo_2_N (8.59 Ω) than Mo_2_C (129.42 Ω) in Table S2. The fast charge transfer process further triggers a superior HER activity on the Mo_2_C-Mo_2_N heterostructure.

DFT calculation reveals that the interfacial atoms, particularly N and Mo atoms, take a pivotal role in determining the high intrinsic activity of the Mo_2_N–Mo_2_C heterostructure. Therefore, more interface sites are created, and more active HER performance will be harvested. Considering a constant total amount of Mo_2_N and Mo_2_C, and only the Mo_2_N content is equal to the Mo_2_C content, could the maximal interfacial abundance possibly be approached. That is why the utmost HER efficiency was observed when the ratio of *n*(Mo-N)/*n*(Mo-C) of Mo_2_C-Mo_2_N@CNWs/D-650 is close to 1, as shown in Figure 4f. In the study by Liu et al., the MoC-Mo_2_C heterostructure was developed as the HER electrocatalyst. They also uncovered that the optimum HER efficiency was attained when the MoC and Mo_2_C exhibited nearly identical concentrations.

Furthermore, 3D-networked CNWs/D possesses a high electrical conductivity, open accessible surface, and large surface area, working as an excellent current collector to efficiently promote the charge transfer on the active sites of porous Mo_2_C-Mo_2_N and enhance the overall high-current HER performance. Figure S16 and Table S2 display that the Mo_2_C-Mo_2_N@CNW_S_/D-650 has a close Tafel slope with the Mo_2_C-Mo_2_N@CC-650, indicating the nearly identical HER kinetics derived from the Mo_2_C-Mo_2_N heterostructure. Nevertheless, owing to more than a two-fold increase in capacitance (also ECSA) stemming from the CNWs/D, the Mo_2_C-Mo_2_N@CNW_S_/D-650 exhibits a much lower overpotential (*η*_1000_ = 194.4 mV) than that of Mo_2_C-Mo_2_N@CC-650 (*η*_1000_ = 251.7 mV).

In short, we constructed a highly active Mo_2_C-Mo_2_N heterointerface and augmented the abundance of interfacial sites through facilely manipulating the annealing temperature. This, coupled with the enhanced ECSA provided by the CNWs/D current collector, synergistically results in exceptional HER performance at high current densities.

## 4. Conclusions

In summary, a binder-free Mo_2_C-Mo_2_N@CNWs/D heterostructure is facilely prepared via ultrasonically soaking in a Mo-salt solution followed by an annealing treatment and the outstanding performance is demonstrated using this self-supporting electrocatalyst in the high-current-density HER. The Mo_2_C-Mo_2_N@CNWs/D not only delivers a low overpotential of 42.8 mV at a small current density of 10 mA/cm^2^ but also maintains impressively low overpotentials of 137.8 and 194.4 mV at high current densities of 500 and 1000 mA/cm^2^, respectively. The outstanding high-current HER performance could be ascribed to synergistic merits derived from the Mo_2_C-Mo_2_N heterostructure and CNWs/D as follows: (1) DFT calculations reveal that the heterojunction of Mo_2_C and Mo_2_N downshifts the d-band center of interfacial Mo orbitals, contributing to a ∆*G*_H*_ value that is close to zero, thus leading to high interfacial activity of Mo_2_C-Mo_2_N. (2) The hetero-interfacial active sites are enriched through elaborately manipulating the proportion of the Mo-C bond to Mo-N bond close to 1. (3) Benefitting from a high electrical conductivity, open accessible surface, and large surface area, the 3D-networked CNWs/D serves as a good current collector to facilitate the electron transfer occurring on the interfacial sites of Mo_2_C-Mo_2_N. This work highlights an effective way to design a highly active non-precious electrocatalyst and promises its applications in the electrochemical hydrogen production industry.

## Figures and Tables

**Table 1:** Electrocatalytic HER characteristics of Mo_2_C-Mo_2_N@CNWs/D-500, -650, -700, -750, -800, and -850. Pt/C@CC is included as control.

Sample	*η*_10_ (mV)	*η*_500_ (mV)	*η*_1000_ (mV)	Tafel Slope (mV/dec)	*R*_ct_ (Ω)	Capacitance (mF/cm^2^)
Mo_2_C-Mo_2_N@CNWs/D-500	83.7	221.5	299.8	54.3	33.94	603
Mo_2_C-Mo_2_N@CNWs/D-650	42.8	137.8	194.4	45.6	8.59	891
Mo_2_C-Mo_2_N@CNWs/D-700	47.9	154.8	207.8	46.5	8.64	359
Mo_2_C-Mo_2_N@CNWs/D-750	62.6	169.4	222.9	48.8	13.95	243
Mo_2_C-Mo_2_N@CNWs/D-800	169.3	369.1	-	97.4	464.65	46
Mo_2_C-Mo_2_N@CNWs/D-850	281.7	502.2	-	109.2	1214.90	40
Pt/C@CC	16.8	228.5	359.3	32.5	-	-

**Table 2:** Comparison of the HER performance of transition-metal-based electrocatalysts in 1.0 M KOH solution.

Catalysts	*η*_10_ (mV)	*η*_500_ (mV)	*η*_1000_ (mV)	Ref.
Mo_2_C-Mo_2_N@CNWs/D-650	42.8	137.8	194.4	This work
Mo_2_C-Mo_2_N/HGr	154	-	-	[21]
Mo*_x_*C	116	-	-	[13]
P-MoP/Mo_2_N	89	-	-	[36]
Mo_2_C/MoC/CNT	82	201	233	[20]
MoS_2_/Mo_2_C	-	191	220	[37]
MoC-Mo_2_C/Mo	98.2	292	-	[19]
Co_2.90_B_0.73_P_0.27_/NF	42	129	165	[38]
Ni_2_P/NF	-	235	306	[39]
Mo_2_N/CoN/NC/Cu	22	155	230	[26]
Ni_2(1−x)_Mo_2x_P/NF	72	240	294	[40]
N-NiMoS/NF	68	250	322	[41]
MoS_2_/Ni_3_S_2_	70	182	200	[33]
P-NiMoHZ	23	175	210	[42]
N-MoO_2_/Ni_3_S_2_ NF	-	431	517	[43]
NiCoP/CF	47	273	379	[44]
Cu-m/Cu–W/NiCo-LDH	21	139	190	[45]
NiCo/NiCo-OH	19	184	-	[46]
A-NiCo LDH/NF	36	286	381	[47]
PrBa_0.94_Co_2_O_5+δ_-DSPH	186	364	-	[48]

## Data Availability

The data presented in this study are available on request from the corresponding author.

## Supplementary Materials

The following supporting information can be downloaded at: https://www.mdpi.com/article/10.3390/nano14030243/s1, Figure S1: Side view (upper panel) and top view (lower panel) for model structures; Figure S2: SEM images of CC; Figure S3: SEM image and Raman spectrum of the CNW/D-coated CC; Figure S4: TEM images of Mo_2_C-Mo_2_N@CNWs/D; Figure S5: EDS spectrum of Mo_2_C-Mo_2_N@CNWs/D; Figure S6: Polarization curves of Mo_2_C-Mo_2_N@CNWs/D prepared at 700 °C with the annealing time; Figure S7: CV curves of Mo_2_C-Mo_2_N@CNWs/D at various scan rates; Figure S8: SEM image of Mo_2_C-Mo_2_N@CNWs/D-650 after a 24 h HER test; Figure S9. Raman spectra of Mo_2_C-Mo_2_N@CNWs/D; Figure S10: The intensity ratio of the Mo_2_C (101) peak to the Mo_2_N (111) peak in XRD patterns as a function of the temperature; Figure S11: XPS survey spectrum of Mo_2_C-Mo_2_N@CNWs/D-650; Figure S12: XPS survey spectrum of Mo_2_C-Mo_2_N@CNWs/D-650 after HER test; Figure S13: High-resolution Mo 3d XPS spectra of Mo_2_C-Mo_2_N@CNWs/D; Figure S14: XRD pattern of Mo_2_C@CNWs/D-650; Figure S15: Electrocatalytic HER performance of Mo_2_C@CNWs/D-650; Figure S16: Electrocatalytic HER performance of Mo_2_C-Mo_2_N@CC-650; Table S1: The adsorption energy change in H^*^ species (∆*E*_H*_) and the free energy change in adsorbed H^*^ species (∆*G*_H*_); Table S2: Electrocatalytic HER characteristics of Mo_2_C@CNWs/D-650, Mo_2_C-Mo_2_N@CC-650, and Mo_2_C-Mo_2_N@CNWs/D-650.
