# Supplementary material for: A Highly Active Porous Mo2C-Mo2N Heterostructure on Carbon Nanowalls/Diamond for a High-Current Hydrogen Evolution Reaction
Source: Nanomaterials (Basel). 2024 Jan 23;14(3):243. doi: 10.3390/nano14030243 (PMC10856447; doi:10.3390/nano14030243)
Supplement: Supplementary file 1 [file nanomaterials-14-00243-s001.zip › nanomaterials-2801513-supplementary.pdf]

Supplementary Materials

A Highly Active Porous Mo_2C-Mo_2N Heterostructure on Carbon Nanowalls/Diamond for a High-current Hydrogen Evolution Reaction

Zhaofeng Zhai [1,2], Chuyan Zhang [1], Bin Chen [1,2], Lusheng Liu [1], Haozhe Song [1], Bing Yang [1,2], Ziwen Zheng [1,2], Junyao Li [1], Xin Jiang [1,3,*] and Nan Huang [1,2,*]

[1] Shenyang National Laboratory for Materials Science (SYNL), Institute of Metal Research (IMR), Chinese Academy of Sciences (CAS), No. 72 Wenhua Road, Shenyang 110016, China
[2] School of Materials Science and Engineering, University of Science and Technology of China, No. 72 Wenhua Road, Shenyang 110016, China
[3] Institute of Materials Engineering, University of Siegen, No. 9-11 Paul-Bonatz-Str., 57076 Siegen, Germany
* Correspondence: xjiang@imr.ac.cn (X.J.); nhuang@imr.ac.cn (N.H.)

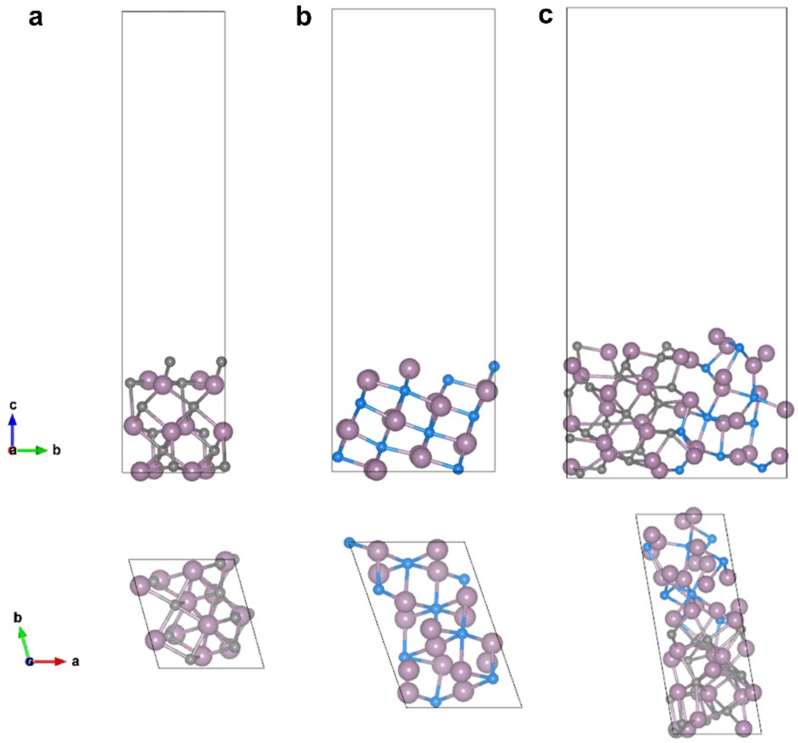

Figure S1. Side view (upper panel) and top view (lower panel) for the super cells of (a) Mo_2C (101), (b) Mo_2N (111), and (c) Mo_2C (101)-Mo_2N (111) heterostructure.

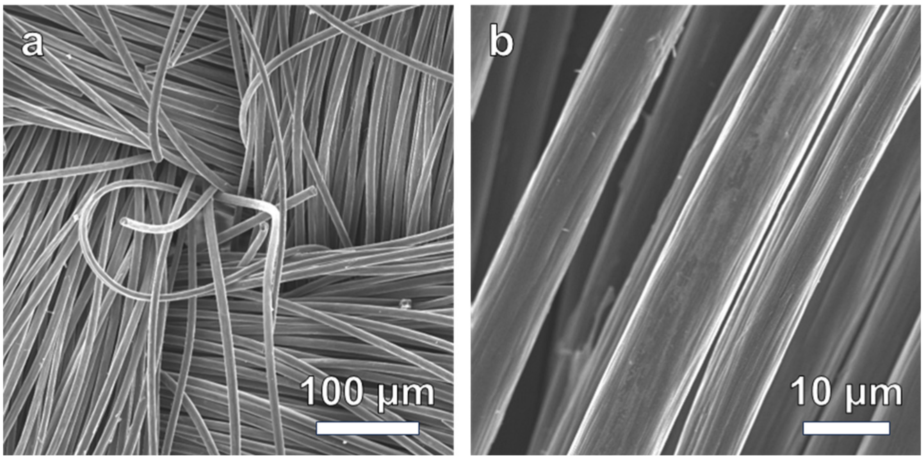

Figure S2. (a) Low-magnification and (b) high-magnification SEM images of CC.

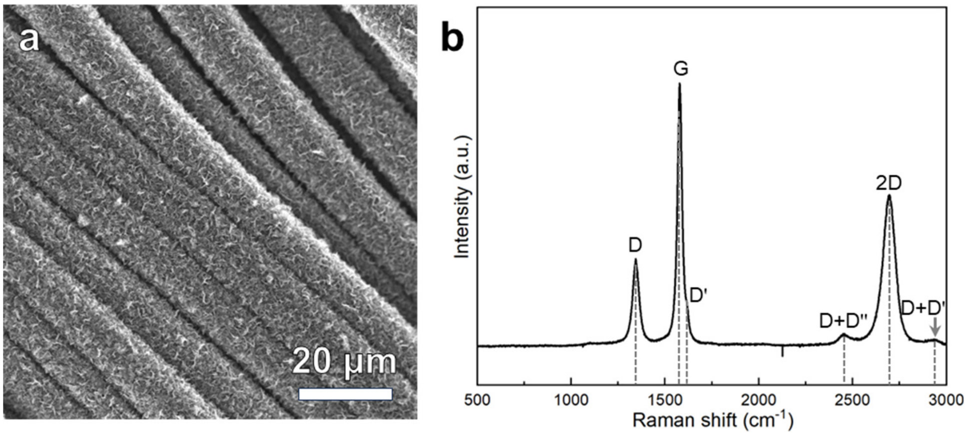

Figure S3. (a) SEM image and (b) Raman spectrum of the pristine CNW/D-coated CC.

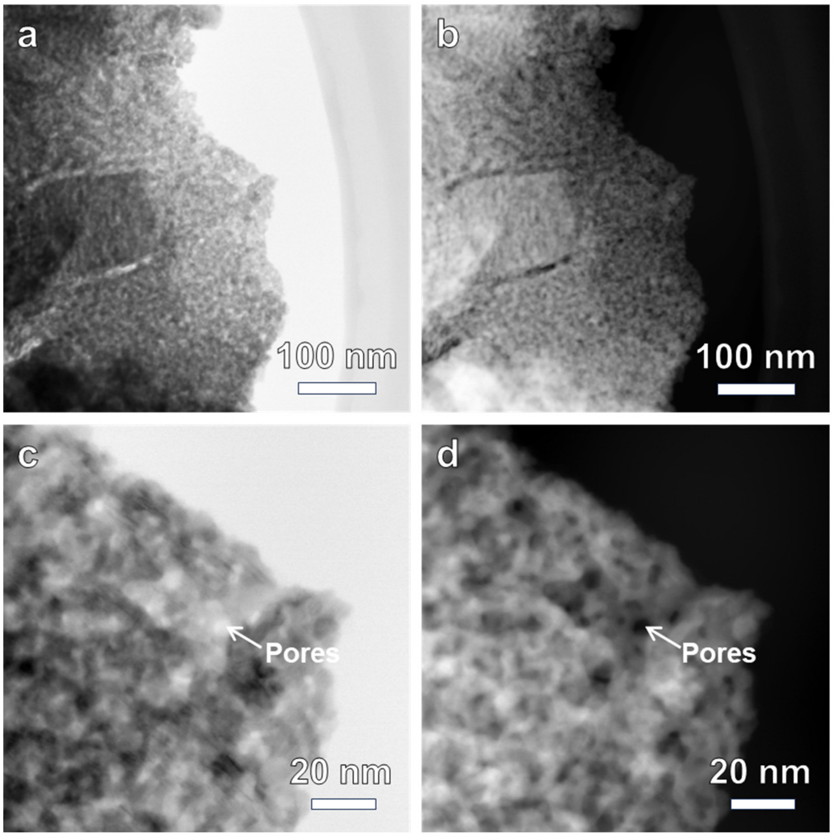

Figure S4. (a,c) Bright-field and (b,d) corresponding HAADF TEM images of Mo_2C-Mo_2N@CNWs/D.

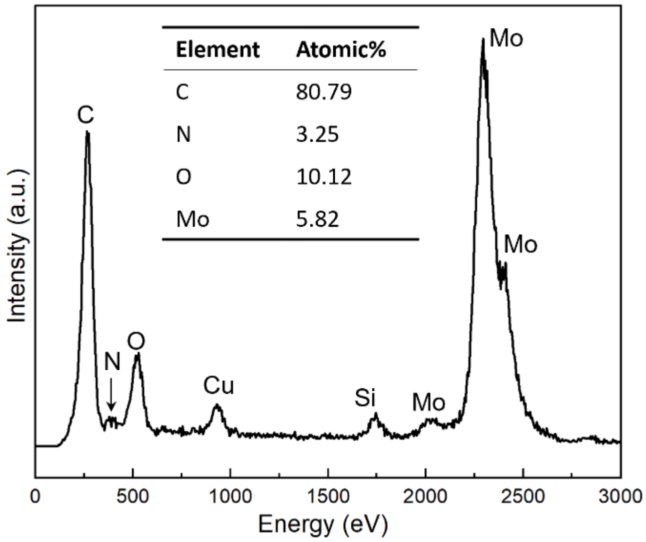

Figure S5. EDS spectrum of Mo_2C-Mo_2N@CNWs/D.

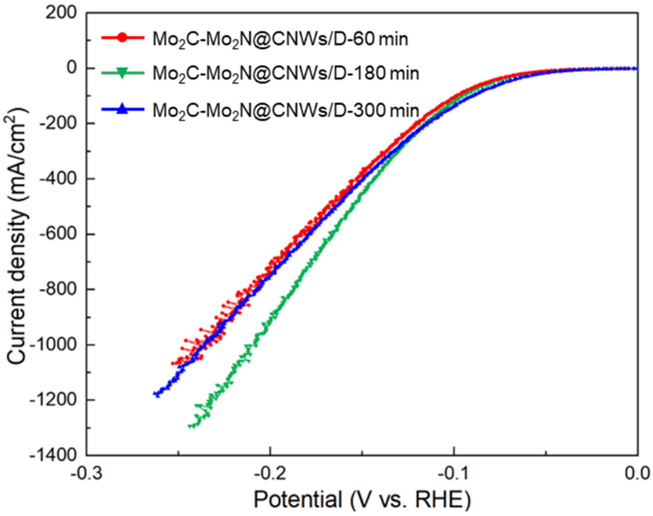

Figure S6. Polarization curves of Mo_2C-Mo_2N@CNWs/D prepared at 700 ºC with the annealing time of 60, 180, and 300 min.

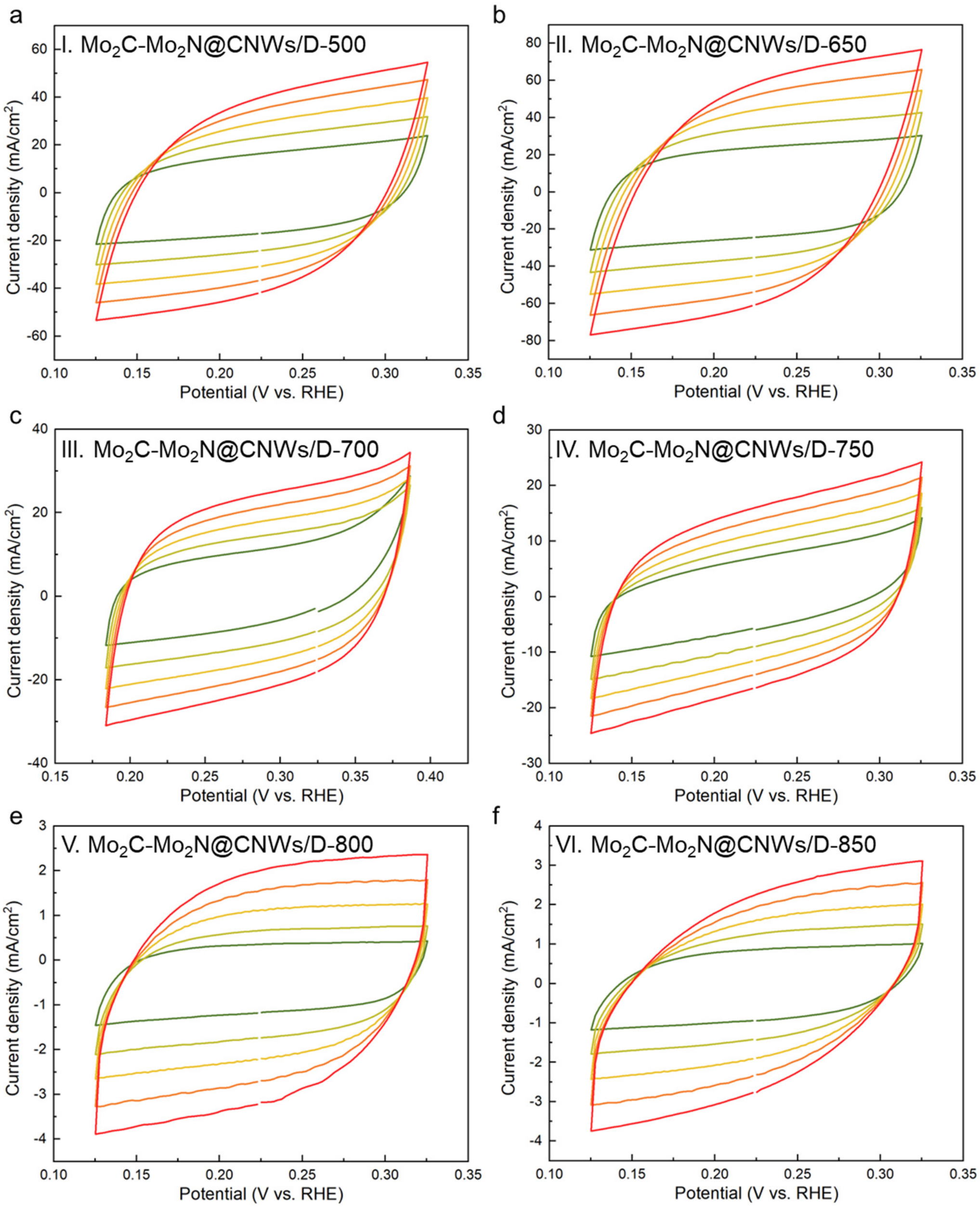

Figure S7. CV curves of (a) Mo_2C-Mo_2N@CNWs/D-500, (b) Mo_2C-Mo_2N@CNWs/D-650, (c) Mo_2C-Mo_2N@CNWs/D-700, (d) Mo_2C-Mo_2N@CNWs/D-750, (e) Mo_2C-Mo_2N@CNWs/D-800, and (f) Mo_2C-Mo_2N@CNWs/D-850 at various scan rates ranging from 0.04 to 0.12 V/s.

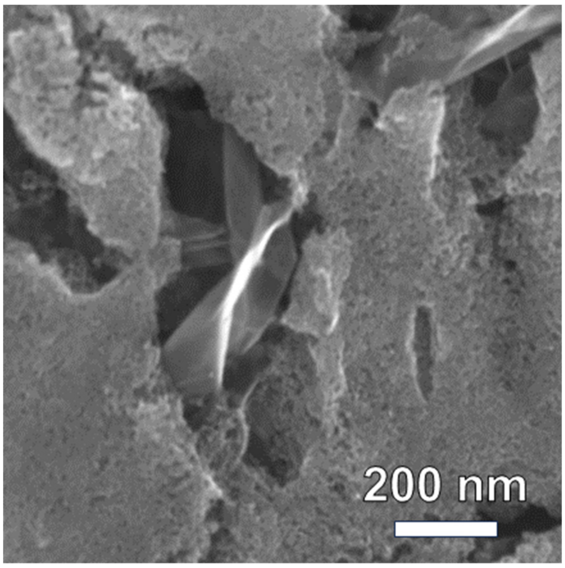

Figure S8. SEM image of Mo_2C-Mo_2N@CNWs/D-650 after a 24 h HER test at a high current density of 500 mA/cm^2.

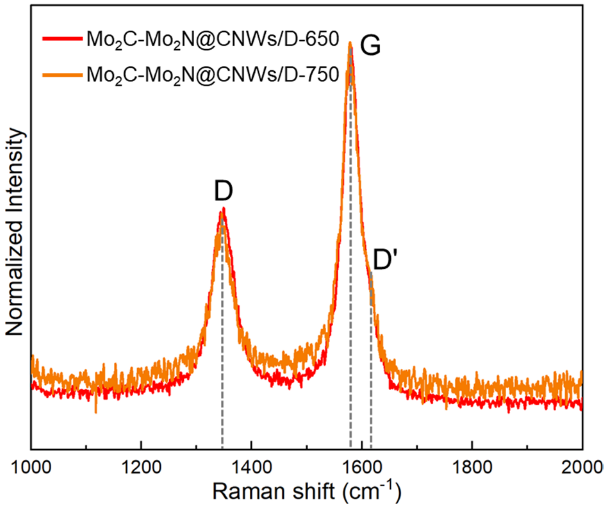

Figure S9. Raman spectra of Mo_2C-Mo_2N@CNWs/D prepared at 650 and 750 ^oC.

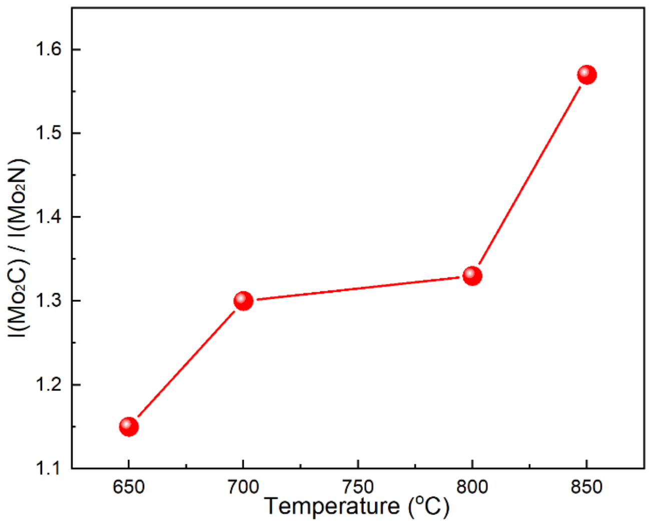

Figure S10. The intensity ratio of the Mo_2C (101) peak to the Mo_2N (111) peak in XRD patterns as a function of the temperature.

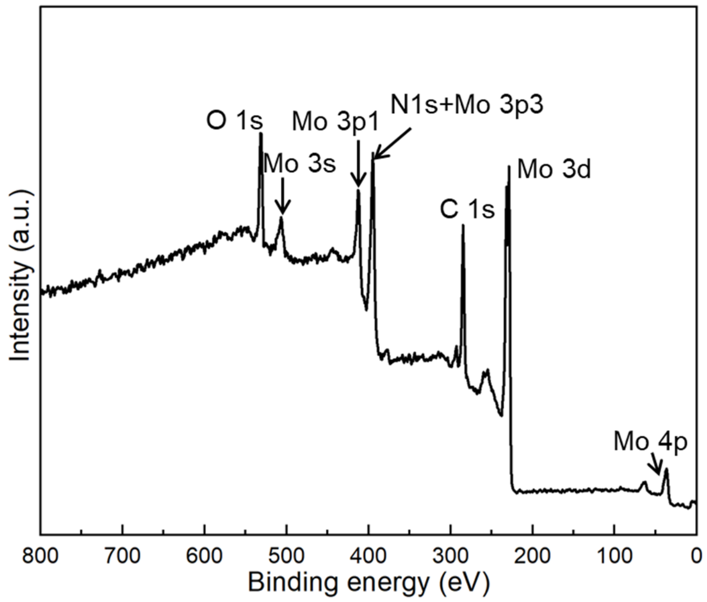

Figure S11. XPS survey spectrum of Mo_2C-Mo_2N@CNWs/D-650.

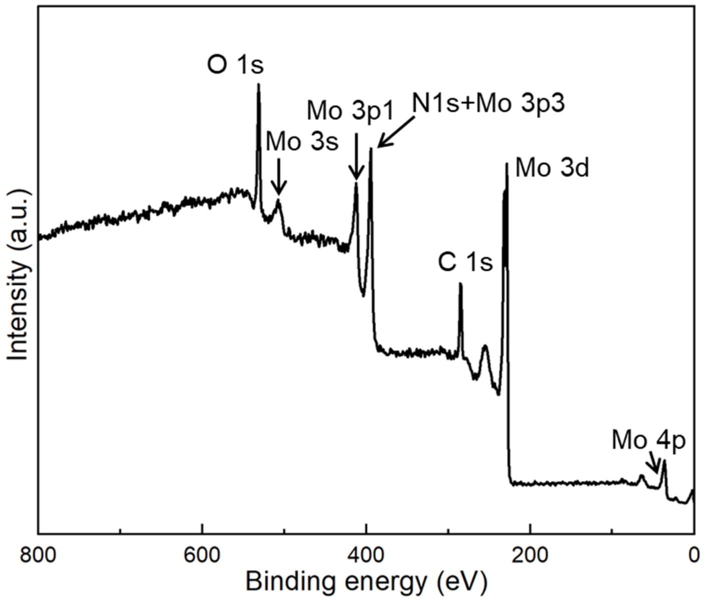

Figure S12. XPS survey spectrum of Mo_2C-Mo_2N@CNWs/D-650 after HER test.

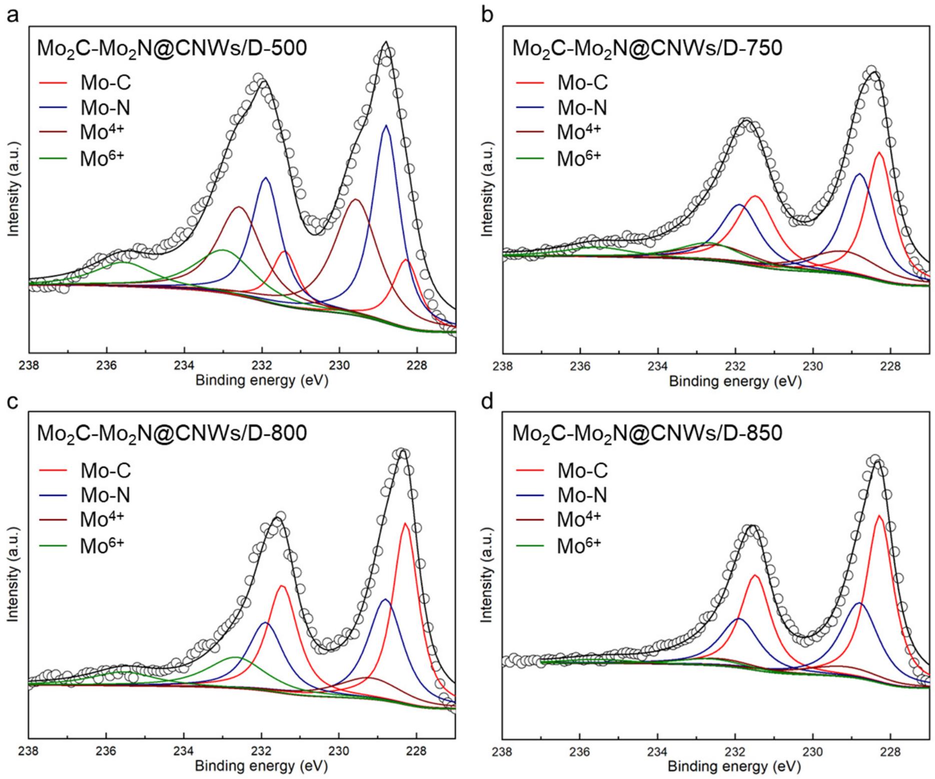

Figure S13. High-resolution Mo 3d XPS spectra of (a) Mo_2C-Mo_2N@CNWs/D-500, (b) Mo_2C-Mo_2N@CNWs/D-750, (c) Mo_2C-Mo_2N@CNWs/D-800, and (d) Mo_2C-

Mo_2N@CNWs/D-850.

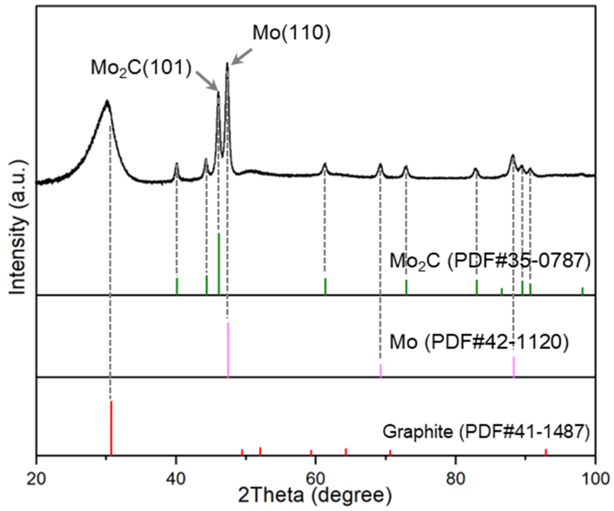

Figure S14. XRD pattern of Mo_2C@CNWs/D-650 prepared on a CC substrate.

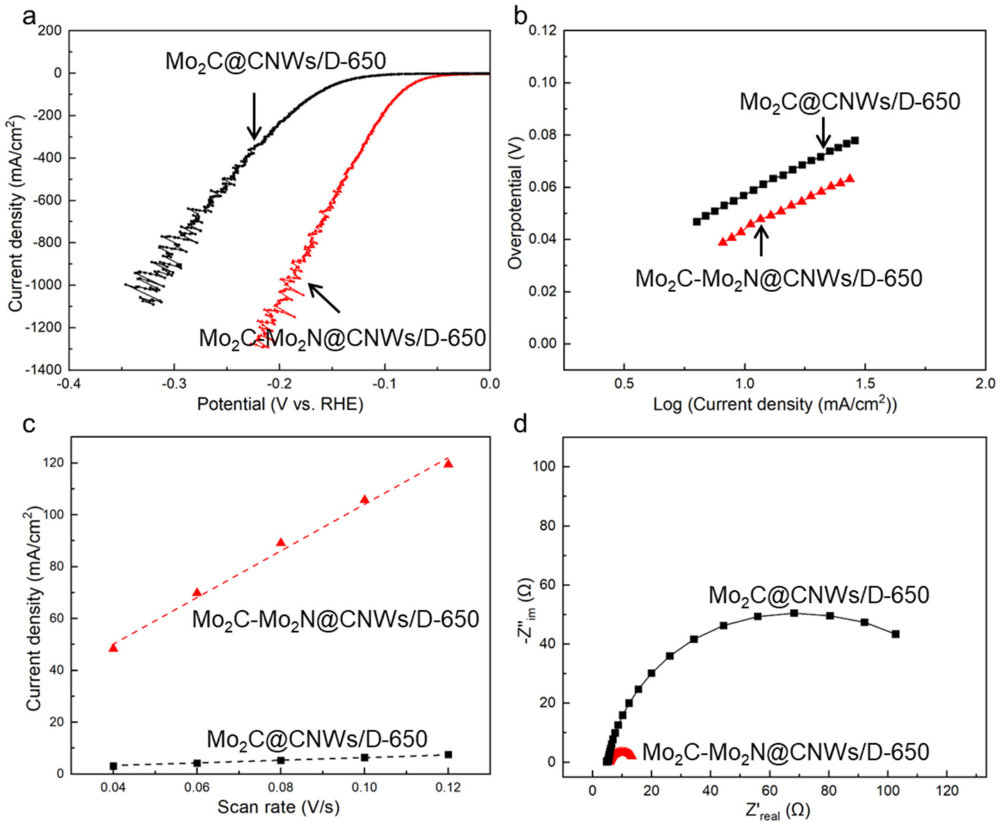

Figure S15. Electrocatalytic HER performance of Mo_2C@CNWs/D-650 in 1 M KOH. (a) Polarization curves and corresponding (b) Tafel plots. (c) Capacitive current

variation as a function of scan rate from 0.04 to 0.12 V/s. (d) Nyquist plots. The curves of Mo_2C-Mo_2N@CNWs/D-650 are demonstrated as control.

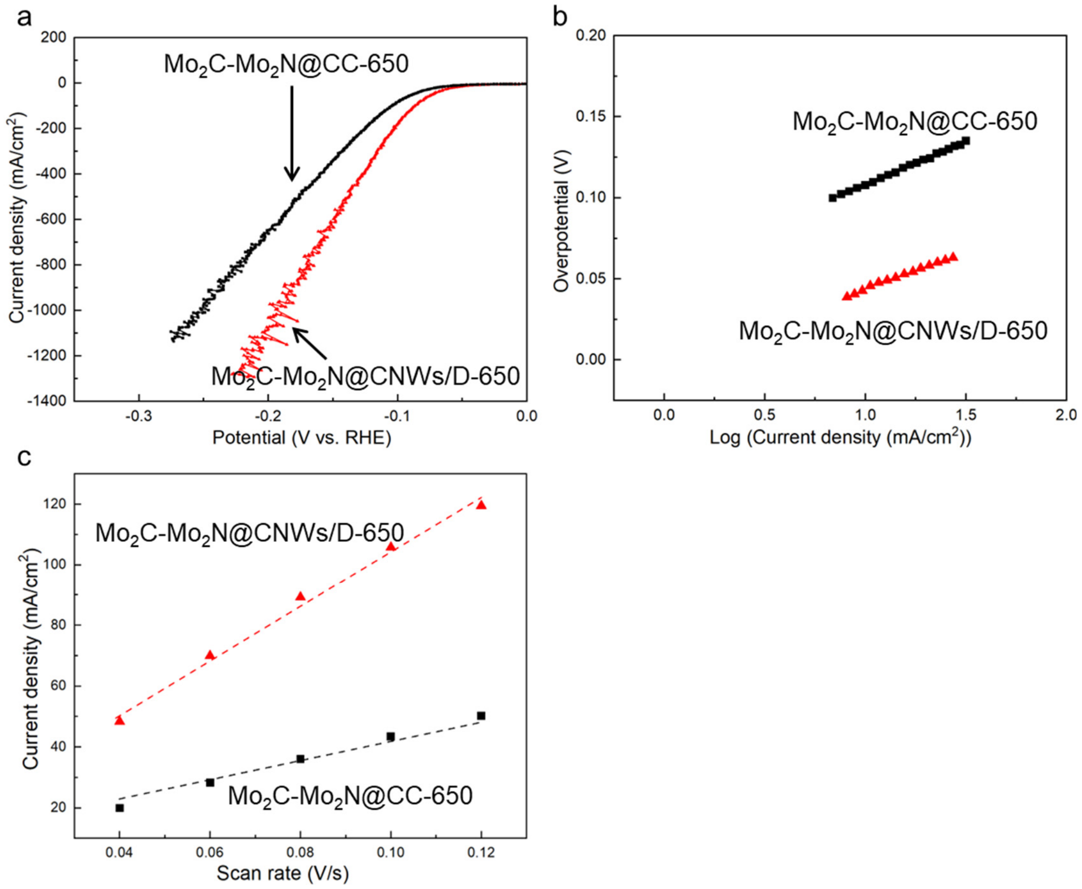

Figure S16. Electrocatalytic HER performance of Mo_2C-Mo_2N@CC-650 in 1 M KOH. (a) Polarization curves and corresponding (b) Tafel plots. (c) Capacitive current variation as a function of scan rate from 0.04 to 0.12 V/s. The curves of Mo_2C-Mo_2N@CNWs/D-650 are demonstrated as control.

Table S1. The adsorption energy change of H^* species (ΔE_{H^*}) and the free energy change of adsorbed H^* species (ΔG_{H^*}) on different sites of Mo_2C (101), Mo_2N (111), and Mo_2C (101)-Mo_2N (111) heterostructure.

**Species**	**ΔE_{H^*} (eV)**	**ΔG_{H^*} (eV)**
H^* on Mo site of Mo_2C	-0.4544	-0.3178
H^* on Mo site of Mo_2N	-0.9586	-0.5889
H^* on Mo site of Mo_2C-Mo_2N	-0.2915	-0.1673
H^* on N site of Mo_2C-Mo_2N	-0.1826	0.0913
H^* on C site of Mo_2C-Mo_2N	-0.9137	-0.6604

Table S2. Electrocatalytic HER characteristics of Mo_2C@CNWs/D-650 prepared without melamine, Mo_2C-Mo_2N@CC-650 prepared on CC, and Mo_2C-Mo_2N@CNWs/D-650.

**Samples**	**η_{10} (mV)**	**η_{500} (mV)**	**η_{1000} (mV)**	**Tafel slope (mV/dec)**	**R_{ct} (Ω)**	**Capacitance (mF/cm^2)**
Mo_2C@CNWs/D-650	107.9	253.8	316.6	52.9	129.42	55
Mo_2C-Mo_2N@CC-650	56.9	176.3	251.7	47.3	13.94	379
Mo_2C-Mo_2N@CNWs/D-650	42.8	137.8	194.4	45.6	8.59	891
